# Adherence rate of quality‐of‐care indicators for *Staphylococcus aureus* bacteremia is extremely low in Japanese emergency and critical care departments: a multicenter retrospective observational study

**DOI:** 10.1002/ams2.316

**Published:** 2017-10-25

**Authors:** Kyohei Miyamoto, Seiya Kato, Junichi Kitayama, Junpei Okawa, Ayana Okamoto, Jun Kamei, Kazuhisa Yoshiya, Hideki Asai, Shingo Adachi, Hidekazu Yukioka, Hiroshi Akimoto, Kazuo Okuchi

**Affiliations:** ^1^ Department of Emergency and Critical Care Medicine Wakayama Medical University Wakayama Japan; ^2^ Emergency and Critical Care Medical Center Osaka Police Hospital Osaka Japan; ^3^ Emergency and Critical Care Center Osaka City General Hospital Osaka Japan; ^4^ Department of Emergency, Disaster and Critical Care Medicine Hyogo College of Medicine Nishinomiya Japan; ^5^ Department of Critical Care Medicine Japanese Red Cross Society Wakayama Medical Center Wakayama Japan; ^6^ Department of Traumatology and Acute Critical Medicine Osaka University Graduate School of Medicine Suita Japan; ^7^ Department of Emergency and Critical Care Medicine Nara Medical University Kashihara Japan; ^8^ Senshu Trauma and Critical Care Center Rinku General Medical Center Izumisano Osaka Japan; ^9^ Department of Anesthesia, Emergency and Intensive Care Yukioka Hospital Osaka Japan; ^10^ Osaka Mishima Emergency Critical Care Center Takatsuki Japan

**Keywords:** Epidemiology, follow‐up blood culture, quality‐of‐care indicator, *Staphylococcus aureus* bacteremia

## Abstract

**Aim:**

*Staphylococcus aureus* bacteremia causes significant morbidity and mortality and requires specific management to prevent complications. Most studies evaluating quality of care have been carried out in Europe and North America, and accurate epidemiological data are lacking in Asia. We aimed to describe the epidemiology and evaluate the quality of care for *S. aureus* bacteremia in Japan.

**Methods:**

From February 2011 to January 2014, we undertook a multicenter retrospective observational study in 10 departments of emergency and critical care in Japan. We included 118 hospitalized adult patients with *S. aureus* bacteremia and evaluated three quality‐of‐care indicators: follow‐up blood culture, treatment duration, and echocardiography.

**Results:**

The mean age of the patients was 63.5 ± 17.0 years. The major source of bacteremia was pneumonia (*n* = 22, 19%), followed by skin and soft tissue infection (*n* = 18, 15%). Thirty patients (25%) died in the hospital. Follow‐up blood culture was performed in 21/112 patients (19%). The duration of antimicrobial treatment was sufficient in 49/87 patients (56%). Echocardiography for patients with clinical indication was undertaken in 39/59 patients (66%). Any of the three indicators were inadequate in 101/118 (86%).

**Conclusion:**

The rate of adequate care for *S. aureus* bacteremia is low in Japan. The low adherence rate for follow‐up blood culture was particularly notable. *Staphylococcus aureus* bacteremia can be an important target of quality improvement interventions.

## Introduction

*STAPHYLOCOCCUS AUREUS* is one of the leading causes of bacteremia. *Staphylococcus aureus* bacteremia (SAB) causes significant morbidity and mortality, and usually causes complications (e.g., infective endocarditis and metastatic abscess). For the prevention and early detection of these complications, US guidelines for methicillin‐resistant *S. aureus* (MRSA) infection recommended several specific management strategies, particularly follow‐up blood cultures, sufficient treatment duration, and echocardiography.^1^ Several observational studies have reported that a higher rate of adherence to these recommendations was associated with lower SAB‐related mortality.^2^, ^3^

Most studies evaluating quality of care have been undertaken in Europe and North America. In Japan, there have been several small observational studies about SAB, involving fewer than 100 patients.^4^, ^5^, ^6^, ^7^ These were all single‐center, rather than multicenter studies. Furthermore, there are few published reports regarding the quality of care for SAB in Asia, and there is a lack of studies on the epidemiology of SAB in Japan.

The aim of our multicenter observational study was to describe the epidemiology and evaluate the quality of care for SAB in Japan.

## Methods

Our study was a multicenter retrospective observational study that was undertaken in 10 departments of emergency and critical care, in 10 different hospitals in Japan. The study design was approved by the institutional review board of Wakayama Medical University (Wakayama, Japan), which waived the requirement for informed consent because of the retrospective and observational nature of the study.

We included patients 20 years of age or older*,* hospitalized between February 2011 and January 2014, with positive blood culture findings for *S. aureus*. We excluded patients with polymicrobial bacteremia, patients whose blood culture results were considered to reflect contamination, and patients who withdrew from therapy at the time of obtaining blood culture.

A previous systematic review reported that six modifiable quality‐of‐care indicators had a significant influence on the prognosis of SAB.^2^ From among these six indicators, we selected three quality‐of‐care indicators in our study: follow‐up blood cultures, treatment duration according to the complexity of infection, and echocardiography in those patients with appropriate clinical indications. The retrospective nature of our study did not allow us to evaluate three further quality‐of‐care indicators: early source control, early use of i.v. cloxacillin for methicillin‐susceptible *S. aureus* (MSSA) as definitive therapy, and adjustment of vancomycin dose according to trough levels. Follow‐up blood cultures were judged adequate if they were obtained within 4 days after the first positive blood cultures. The rate of adequate follow‐up blood cultures was calculated as the number of patients with adequate follow‐up blood cultures / the number of patients alive at 96 h. Treatment duration according to the complexity of infection was judged adequate if the duration of antimicrobial therapy was ≥14 days for uncomplicated bacteremia and ≥28 days for complicated bacteremia. The rate of adequate treatment duration was calculated as the number of patients with appropriate duration of therapy / the number of patients alive at 14 or 28 days in cases of uncomplicated or complicated bacteremia, respectively.

Echocardiography in patients with clinical indications was judged adequate if echocardiography was carried out in patients with complicated bacteremia, irrespective of the type of echocardiography (transthoracic or transesophageal). The rate of adequate echocardiography was calculated as the number of patients with echocardiography / the number of patients with complicated bacteremia. Uncomplicated bacteremia was defined according to the Clinical Practice Guidelines of the Infectious Disease Society of America for the treatment of MRSA infections.^1^ We regarded the cases as uncomplicated bacteremia if they met all of the following criteria: exclusion of endocarditis, absence of implanted prostheses, negative follow‐up blood cultures, defervescence within 72 h of therapy, and absence of evidence of metastatic sites of infection. When we judged the complexity, we regarded the cases in which no follow‐up blood culture was performed as negative follow‐up blood cultures. We defined the cases that did not adhere to any of the three quality‐of‐care indicators as “non‐adherent patients.” We defined the other cases as “adherent patients.” The severity of disease was evaluated at the first positive blood culture, as determined using the Acute Physiology And Chronic Health Evaluation II (APACHE II) score and the Pitt bacteremia score.^8^ We collected data about the source of bacteremia from medical records. We did not use any specific diagnostic criteria for identifying the source of bacteremia.

Patients’ characteristics and the quality‐of‐care indicators were compared between survivors and non‐survivors. We also compared the patients’ characteristics between adherent patients and non‐adherent patients.

### Statistical analysis

Continuous variables were presented as mean ± standard deviation or median and interquartile range. Categorical variables are presented as number (%). Two groups were compared using the *t*‐test or the Wilcoxon rank‐sum test for continuous variables and Fisher's exact test for categorical variables. A two‐sided *P*‐value of <0.05 was considered statistically significant, and all analyses were undertaken using JMP Pro software (version 12.2; SAS Institute, Cary, NC, USA).

## Results

During the 3‐year study period, 118 patients with SAB were identified. The patients’ characteristics are presented in Table 1. The major source of bacteremia was pneumonia (19%), followed by skin and soft tissue infection (15%), and osteoarticular infection (13%). The rate of cases with pneumonia varied greatly across institutions, from 0% to 50%. In particular, two institutes experienced 10 cases of pneumonia (45%), out of 22 cases of pneumonia overall, across all institutes. Non‐survivors were older and had higher APACHE II scores than those of survivors.

**Table 1:** Characteristics of 118 hospitalized adult patients with *Staphylococcus aureus* bacteremia

Characteristics	All patients (*n* = 118)	Survivors (*n* = 88)	Non‐survivors (*n* = 30)	*P*‐value
Age, year, mean ± SD	63.6 ± 17.7	60.5 ± 18.2	72.5 ± 12.7	0.0012
Male, *n* (%)	82 (69)	64 (72)	18 (60)	0.2500
APACHE II score, mean ± SD^a^	19.8 ± 9.9	17.6 ± 8.6	26.4 ± 10.7	<0.0001
Pitt bacteremia score, median (IQR)^b^	2 (1–4)	2 (1–4)	4 (1–7)	0.0270
Comorbidity				
Immunosuppression, *n* (%)	14 (12)	9 (10)	5 (17)	0.3400
Chronic dialysis, *n* (%)	7 (6)	6 (7)	1 (3)	0.6800
Home oxygenation therapy, *n* (%)	4 (3)	3 (3)	1 (3)	1.0000
Liver cirrhosis, *n* (%)	3 (3)	1 (1)	2 (7)	0.1600
Decompensated heart failure, *n* (%)	3 (3)	2 (2)	1 (3)	1.0000
Acquisition				0.2300
Community‐acquired infection, *n* (%)	32 (27)	26 (30)	6 (20)	
Health‐care‐related, community‐acquired infection, *n* (%)	12 (10)	6 (7)	6 (20)	
Hospital‐acquired infection, *n* (%)	42 (36)	32 (36)	10 (33)	
Unclassified, *n* (%)^c^	32 (27)	24 (27)	8 (27)	
Source of bacteremia				0.9200
Pneumonia, *n* (%)	22 (19)	16 (18)	6 (20)	
Skin and soft tissue infection, *n* (%)	18 (15)	15 (17)	3 (10)	
Osteoarticular infection, *n* (%)	15 (13)	12 (14)	3 (10)	
Deep‐seated abscess, *n* (%)	11 (9)	8 (9)	3 (10)	
Urinary tract infection, *n* (%)	9 (8)	7 (8)	2 (7)	
Catheter‐related bloodstream infection, *n* (%)	9 (8)	7 (8)	2 (7)	
Infective endocarditis, *n* (%)	3 (3)	3 (3)	0 (0)	
Others, *n* (%)	9 (8)	6 (7)	3 (10)	
Unknown, *n* (%)	22 (19)	14 (16)	8 (27)	
Prosthetic device infection, *n* (%)^d^	17 (14)	16 (21)	1 (3)	0.0370
Complicated bacteremia, *n* (%)	62 (53)	46 (52)	16 (53)	1.0000
Metastatic infection, *n* (%)	23 (19)	19 (22)	4 (13)	0.4300
Persistent fever (>72 h), *n* (%)	34 (29)	25 (29)	9 (30)	1.0000
Positive follow up blood culture, *n* (%)	14 (12)	8 (9)	6 (20)	0.1900
Implanted prosthesis, *n* (%)	13 (11)	10 (13)	3 (10)	1.0000
Resistance to methicillin				0.7500
Methicillin susceptible, *n* (%)	64 (54)	49 (56)	15 (50)	
Methicillin resistant, *n* (%)	53 (45)	38 (43)	15 (50)	
Unknown, *n* (%)	1 (1)	1 (1)	0 (0)	

a Acute Physiology and Chronic Health Evaluation II (APACHE II) score was evaluated from the data obtained within 24 h after the first positive blood culture drawing. APACHE II scores were missing in five patients.

b Pitt bacteremia score was missing in one patient.

c Patients who were transferred from other hospitals were regarded as “unclassified” because of missing data.

d Infective prosthetic devices, including central venous catheters, were removed in all patients. The timing of removal was within 2 weeks in 14 patients and after 2 weeks in three patients.

IQR, interquartile range; SD, standard deviation.

Methicillin‐resistant *S. aureus* was identified in 53 patients (45%). For these patients, anti‐MRSA agents, including vancomycin, teicoplanin, linezolid, and daptomycin, were prescribed on the day of the first positive blood culture in 22 patients (42%) and the day after proof of MRSA was obtained in 25 patients (47%). Anti‐MRSA agents were not prescribed in the remaining six patients (11%). The first‐choice anti‐MRSA agents used included vancomycin in 28 patients (60%), teicoplanin in 11 patients (23%), linezolid in six patients (13%), and daptomycin in two patients (4%).

Methicillin‐susceptible *S. aureus* was identified in 64 patients (54%). For these patients, antibiotics to which MSSA was susceptible were prescribed on the day of the first positive blood culture in 62 patients (97%) and on the day after proof of MSSA was prescribed in one patient. Antibiotics to which MSSA was susceptible were not prescribed in the remaining patient.

The adherence to quality‐of‐care indicators are presented in Table 2. One‐hundred‐and‐one patients (86%) were non‐adherent to any of the three indicators. The rate of adherence to these indicators was not statistically different between survivors and non‐survivors. We compared patients’ characteristics between adherent patients and non‐adherent patients and found that milder patients were prone to low quality of care (Table S1). Fourteen patients (12%) were complicated with persistent bacteremia. Thirty patients (25%) died in hospital. Median length of hospital stay was 38 (16–76) days.

**Table 2:** Adherence to quality‐of‐care indicators and clinical outcomes in 118 hospitalized adult patients with *Staphylococcus aureus* bacteremia

	All patients (*n* = 118)	Survivors (*n* = 88)	Non‐survivors (*n* = 30)	*P*‐value
Quality‐of‐care indicator, *n* (%)				
Follow‐up blood culture	21/112 (19)	17/86 (20)	4/26 (15)	0.78
Treatment duration	49/87 (56)	40/71 (56)	9/16 (56)	1.00
Echocardiography	39/59 (66)	31/45 (69)	8/14 (57)	0.52
Non‐adherent to any of three indicators	101/118 (86)	76/88 (86)	25/30 (83)	0.76

## Discussion

In our study, the in‐hospital mortality rate of SAB was 25%. Adherence to the three quality‐of‐care indicators was generally low (19–66%) and, in most patients, adherence to any of the three indicators was considered inadequate.

Previous observational studies have reported a similar mortality rate for SAB (15–40%) as in our study.^2^, ^3^, ^4^, ^5^, ^6^, ^9^, ^10^ However, the sources of bacteremia differed significantly between studies. In our study, the most common source of bacteremia was pneumonia (19%). Previous Japanese observational studies reported an inconsistent rate of pneumonia. One observational study reported a similar rate of pneumonia (17/83, 21%)^6^ as that reported in the present study, but another study reported a lower rate of pneumonia (4/73, 5%).^7^ In contrast, the studies of SAB that were undertaken outside of Japan generally reported a low rate of pneumonia.^9^, ^10^ A prospective observational study of SAB in the USA reported a low rate of respiratory tract infection (12/505, 2%).^9^ Another prospective study in Spain also reported a low rate of respiratory tract infection (64/908; 7%).^10^ In our study, specific diagnostic criteria for pneumonia were not available, and the rate of the cases with pneumonia varied greatly between institutions. In particular, only two institutes accounted for almost half of all cases of pneumonia. This may suggest that the surprisingly high incidence of the pneumonia in our study is simply biased by the attending physicians’ decision, and does not reflect the true incidence of pneumonia. *Staphylococcus aureus* pneumonia may be overdiagnosed in Japan.

In our study, the adherence rate of three quality‐of‐care indicators was generally low, and the adherence rate of follow‐up blood culture was particularly low (19%). In two previous observational studies outside of Asia, the adherence rates of follow‐up blood culture were better than in our study (31%^3^ and 61%^2^). These adherence rates were improved by interventions, including infectious diseases service consultation (41%^3^ and 80%^2^), and this improvement was associated with better survival.^2^, ^3^ Furthermore, observational studies have reported that infectious disease service consultation was independently associated with reduced SAB‐related mortality.^3^, ^11^ Similarly, an observational study has reported that routine infectious disease service consultation for positive blood cultures resulted in improved detection of infective endocarditis.^12^ Routine infectious disease service consultation thus seems to be a promising strategy for managing SAB. In contrast, adherence to these indicators was not associated with survival in our study, but this result was not inconsistent with previous studies. The adherence to indicators per se cannot improve outcomes; rather, the interventions related to adherence to these indicators improve outcomes.

Our study showed that adequate management for SAB is rare in Japanese emergency and critical care departments. There is much room for improvement of SAB management in Japan, especially in milder patients.

However, our study had several limitations. First, it was a retrospective study. Although the above‐mentioned systematic review reported six quality‐of‐care indicators,^2^ we could collect data for only three indicators, due to the retrospective nature of our study. A prospective study is needed to evaluate all six quality‐of‐care indicators. However, we were able to show that the rate of adherence to at least these three indicators is extremely low in Japan. Second, our study focused on a particular context: emergency and critical care departments, which may limit extrapolation of our results to other circumstances. Third, our study included a relatively smaller number of patients than those in other observational studies that have been carried out in general hospitalized patients.^9^, ^10^ This might be because our study included patients hospitalized only in emergency and critical care departments, and not all hospitalized patients. Another reason might be that many cases of SAB remain undiagnosed in emergency and critical care departments in Japan; however, the retrospective nature of our study does not allow the testing of these hypotheses. Future prospective studies are needed in general hospitalized patients with SAB, to evaluate the effectiveness of interventions for quality‐of‐care indicators and the recognition rate of SAB in Japan.

## Conclusions

In‐hospital SAB‐related mortality affected one‐quarter of patients. The rate of adequate care for SAB in Japanese emergency and critical care departments was extremely low. The low adherence rate of follow‐up blood culture was especially notable. *Staphylococcus aureus* bacteremia should be an important target for quality improvement interventions.

## Disclosure

Approval of the research protocol: The protocol for this research project was approved by a suitably constituted Ethics Committee of the institution and it conforms to the provisions of the Declaration of Helsinki (Committee of Wakayama Medical University, Approval No. 1414). It waived the requirement for informed consent because of the retrospective and observational nature of the study.

Registry and the registration no. of the study/trial: This study was not registered.

Conflict of interest: None.

## Supporting information


**Table S1.** Comparison of main characteristics of patients with *Staphylococcus aureus* bacteremia by the adherence to quality‐of‐care indicators
