# Supplementary material for: Adherence rate of quality‐of‐care indicators for Staphylococcus aureus bacteremia is extremely low in Japanese emergency and critical care departments: a multicenter retrospective observational study
Source: Acute Med Surg. 2017 Oct 25;5(2):140–5. doi: 10.1002/ams2.316 (PMC5891101; doi:10.1002/ams2.316)
Supplement: Supplementary file 1 — Table S1. Comparison of main characteristics of patients with Staphylococcus aureus bacteremia by the adherence to quality‐of‐care indicators [file AMS2-5-140-s001.docx]

Supplementary Table. Comparison of main patients' characteristics by the adherence to quality-of-care indicators

| Characteristics | Adherent patients † (n = 17) | Non-adherent patients † (n = 101) | *P* value |
| --- | --- | --- | --- |
| Age, y, mean ± SD | 58.7 ± 15.2 | 64.4 ± 18.0 | 0.22 |
| Male, number (%) | 11 (65) | 71 (70) | 0.78 |
| APACHE II score, mean ± SD ‡ | 25.2 ± 10.7 | 18.9 ± 9.5 | 0.018 |
| Pitt bacteremia score, median (IQR) § | 4 (2–8) | 2 (1–4) | 0.057 |
| Source of bacteremia |  |  | 0.020 |
| Pneumonia, number (%) | 2 (12) | 20 (20) |  |
| Skin and soft tissue infection, number (%) | 0 (0) | 18 (18) |  |
| Osteoarticular infection, number (%) | 4 (24) | 11 (11) |  |
| Deep-seated abscess, number (%) | 3 (18) | 8 (8) |  |
| Urinary tract infection, number (%) | 0 (0) | 9 (9) |  |
| Catheter related blood stream infection, number (%) | 0 (0) | 9 (9) |  |
| Infective endocarditis, number (%) | 2 (12) | 1 (1) |  |
| Others, number (%) | 1 (6) | 8 (8) |  |
| Unknown, number (%) | 5 (29) | 17 (17) |  |
| Resistance to methicillin |  |  | 0.31 |
| Methicillin susceptible, number (%) | 12 (71) | 52 (51) |  |
| Methicillin resistant, number (%) | 5 (29) | 48 (48) |  |
| Unknown, number (%) | 0 (0) | 1 (1) |  |

† We defined the cases being non-adherent to any of three quality-of-care indicators as "non-adherent". We defined the other cases as "adherent".

‡ APACHE II score was evaluated from the data obtained within 24 h after the first positive blood culture drawing. APACHE II scores were missing in five patients.

§ Pitt bacteremia score was missing in one patient.

SD: Standard deviation, APACHE II: Acute physiology and chronic health evaluation II, IQR: Interquartile range
